# Mitochondrial UQCRC2 as a Redox-Regulatory Node in Metabolic and Cardiometabolic Diseases

**DOI:** 10.3390/antiox15070794

**Published:** 2026-06-25

**Authors:** Shiyi Chen, Yang Jiao, Wen Shen, Xingru Hu, Guoyue Yuan, Jue Jia

**Affiliations:** 1 Department of Endocrinology and Metabolism, The Affiliated Hospital of Jiangsu University, Zhenjiang 212001, China; 2 Institute of Endocrine and Metabolic Diseases, Jiangsu University, Zhenjiang 212001, China

**Keywords:** UQCRC2, complex III, mitochondrial ROS, redox imbalance, oxidative stress, insulin resistance

## Abstract

Metabolic and cardiometabolic diseases are closely associated with mitochondrial dysfunction and redox imbalance. Ubiquinol–cytochrome c reductase core protein 2 (UQCRC2), a non-catalytic structural core subunit of mitochondrial respiratory chain Complex III, is increasingly recognized as a regulator of Complex III integrity, electron transfer, oxidative phosphorylation, and mitochondrial redox homeostasis. Under metabolic stress, reduced expression or functional impairment of UQCRC2 may promote electron leakage, mitochondrial reactive oxygen species (mtROS) generation, lipid peroxidation, impaired antioxidant defense, and disrupted glucose–lipid metabolism. These alterations may contribute to insulin resistance (IR), metabolic dysfunction-associated steatotic liver disease (MASLD), obesity, and cardiovascular disease (CVD). This review summarizes current evidence linking UQCRC2 dysfunction to mitochondrial bioenergetic failure, oxidative stress, inflammatory signaling, and cardiometabolic injury. We further discuss redox-regulatory pathways, including Nrf2, AMPK–SIRT1–PGC-1α, glutathione metabolism, and mitophagy, as well as pharmacological agents and natural compounds that may modulate UQCRC2-related mitochondrial responses. Collectively, these findings highlight UQCRC2 as a redox-sensitive mitochondrial node linking Complex III dysfunction to cardiometabolic injury and targeted redox-based interventions.

## 1. Introduction

Metabolic and cardiometabolic diseases, including type 2 diabetes mellitus (T2DM), obesity, metabolic dysfunction-associated steatotic liver disease (MASLD), and metabolic syndrome (MS), have emerged as major global health challenges. Driven by rapid alterations in lifestyle patterns and population aging, their prevalence continues to rise sharply [1]. Current estimates suggest that more than 828 million adults worldwide are living with diabetes, while MASLD affects approximately 38% of the global population, making it the most prevalent chronic liver disease [2]. Beyond their widespread prevalence, these disorders markedly increase the risk of cardiovascular, cerebrovascular, and hepatic complications, ultimately impairing quality of life and imposing substantial socioeconomic burdens.

The pathogenesis of metabolic diseases is multifactorial, with insulin resistance (IR) as a central feature. Driving factors, including disordered lipid metabolism, chronic low-grade inflammation, oxidative stress, and impaired β-cell function, interact to accelerate disease progression [3]. Notably, these heterogeneous processes converge on mitochondrial dysfunction, which provides a common mechanistic nexus for downstream organ injury. Mitochondria are central to energy homeostasis through oxidative phosphorylation (OXPHOS), fatty acid oxidation, and the tricarboxylic acid (TCA) cycle. However, mitochondrial dysfunction in metabolic diseases is not merely an energetic defect, but also a redox disorder characterized by excessive mitochondrial reactive oxygen species (mtROS) generation, lipid peroxidation, impaired antioxidant buffering, and activation of inflammatory signaling pathways. Within the mitochondrial respiratory chain, impaired Complex III activity can disrupt glucose and lipid metabolism, increase electron leakage, enhance mtROS production, and trigger oxidative stress, thereby contributing to the onset and progression of T2DM, MASLD, obesity, and cardiovascular complications [4]. Overall, mitochondrial dysfunction, especially at Complex III, appears to serve as a mechanistic bridge between metabolic dysregulation and systemic disease.

Among the 11 subunits of Complex III, ubiquinol-cytochrome c reductase core protein II (UQCRC2) is essential for maintaining structural stability and bioenergetic function [5]. By supporting Complex III integrity and efficient electron transfer, UQCRC2 contributes to OXPHOS, ATP production, and mitochondrial redox homeostasis. Conversely, reduced expression or functional impairment of UQCRC2 may compromise Complex III activity, promote electron leakage, increase mtROS generation, and aggravate oxidative stress [6]. Although studies of UQCRC2 in metabolic disorders remain limited, current evidence suggests that UQCRC2 dysfunction may impair mitochondrial performance and contribute to disease pathogenesis.

Therefore, this review provides a narrative overview of current knowledge of UQCRC2 in metabolic and cardiometabolic diseases. We outline its role in mitochondrial function, bioenergetics, and redox homeostasis, and synthesize evidence from experimental, translational, and emerging clinical studies linking UQCRC2 to T2DM, MASLD, obesity, and cardiovascular disease. We further discuss its potential as a biomarker and therapeutic target, together with key directions for future research, clinical translation, and redox-based therapeutic strategies.

## 2. Biological Features and Basic Functions of UQCRC2

### 2.1. Structural and Tissue Localization of UQCRC2

UQCRC2, as a noncatalytic structural scaffold of complex III, maintains the essential geometry of the Qo and Qi sites, thereby ensuring efficient operation of the Q-cycle and its coupling to OXPHOS [7]. At the tissue level, UQCRC2 expression generally reflects mitochondrial oxidative capacity. High UQCRC2 expression in the heart is consistent with the dependence of cardiomyocytes on OXPHOS. In skeletal muscle, UQCRC2 may be more functionally relevant in mitochondria-rich oxidative fibers, such as type I and type IIa fibers, than in fast glycolytic fibers, which contain fewer mitochondria and rely predominantly on glycolytic metabolism. In contrast, although white adipose tissue shows relatively lower basal UQCRC2 expression, its metabolic function may still be sensitive to UQCRC2-related redox imbalance because mitochondrial redox changes can affect adipocyte differentiation, inflammatory remodeling, and insulin sensitivity [8,9].

**Figure 1:**
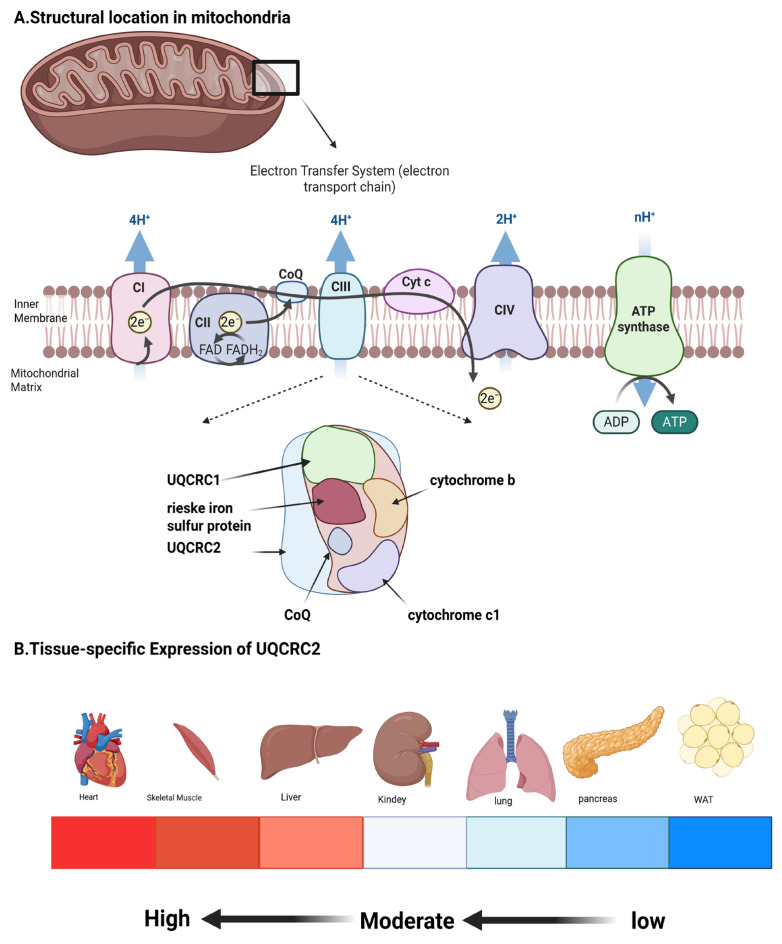
Structural localization and tissue-specific expression of UQCRC2. (**A**) UQCRC2 is localized to the mitochondrial inner membrane as a core structural subunit of respiratory chain Complex III. (**B**) Relative tissue expression profile of UQCRC2 across major organs, showing higher levels in energy-demanding tissues (heart and skeletal muscle) and lower levels in white adipose tissue (WAT); the color scale indicates expression from low (blue) to high (red). Black arrows indicate electron flow or structural annotations, blue arrows indicate proton translocation and ATP synthesis, dashed arrows indicate the enlarged view of Complex III, and bottom gradient arrows indicate relative UQCRC2 expression across tissues. Created in BioRender. Chen, S. (2026) https://BioRender.com/5posp65, accessed on 10 June 2026.

Collectively, it suggests that the impact of UQCRC2 dysfunction depends not only on basal tissue expression, but also on mitochondrial oxidative demand and sensitivity to redox stress [10] (Figure 1). 

### 2.2. Core Bioenergetic and Redox Functions of UQCRC2

**Figure 2:**
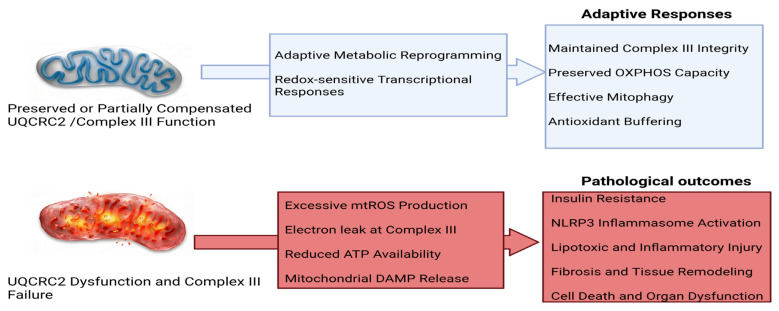
UQCRC2-dependent stress responses and downstream outcomes. Preserved UQCRC2/Complex III function supports adaptive mitochondrial responses, including efficient electron transfer, ATP generation, controlled ROS signaling, antioxidant buffering, and mitochondrial quality control, whereas UQCRC2 dysfunction drives electron leak, mtROS excess, ATP loss and DAMP release, leading to IR, inflammasome activation, inflammatory/fibrotic injury, and organ dysfunction. Created in BioRender. Chen, S. (2026) https://BioRender.com/3uixj9v, accessed on 10 June 2026.

UQCRC2 sustains mitochondrial energy metabolism by stabilizing Complex III architecture and its coupling to OXPHOS. Functionally, it promotes efficient electron transfer and coupling, thereby supporting mitochondrial ATP synthesis in energy-demanding cells. At the same time, by limiting abnormal electron leakage from complex III, UQCRC2 regulates mitochondrial redox balance and prevents excessive accumulation of mtROS [11,12]. When the expression or function of UQCRC2 is disrupted, the efficiency of Complex III decreases, and redox homeostasis becomes vulnerable, making cells more prone to metabolic dysfunction (Figure 2). In this figure, adaptive mitochondrial responses refer to protective processes supported by preserved UQCRC2/Complex III function, including maintained Complex III integrity, preserved OXPHOS capacity, effective mitophagy and antioxidant buffering.

### 2.3. UQCRC2 as a Redox-Sensitive Node Within Mitochondrial Complex III

UQCRC2 should not be viewed as the only regulatory subunit of mitochondrial Complex III or as a catalytic redox enzyme. Rather, its importance lies in its role as a structural core component that helps maintain Complex III integrity, electron-transfer efficiency, and Q-cycle coupling to OXPHOS. Impaired UQCRC2 expression or function may destabilize Complex III, leading to reduced electron-transfer efficiency. This result promotes electron leakage and mtROS generation, ultimately increasing oxidative stress and disrupting mitochondrial redox homeostasis [6].

In this context, UQCRC2 can be considered a redox-sensitive mitochondrial node. Changes in its abundance, structural integrity, or assembly state may indicate impaired Complex III stability under metabolic stress, which can promote mtROS accumulation and downstream metabolic or inflammatory injury. This view does not exclude the contribution of other Complex III subunits, such as UQCRC1, UQCRH, or CYC1, but highlights UQCRC2 as a structurally defined contributor to Complex III-centered redox instability in metabolic and cardiometabolic diseases.

## 3. UQCRC2-Dependent Mitochondrial Redox Dysfunction in Metabolic and Cardiometabolic Diseases

**Figure 3:**
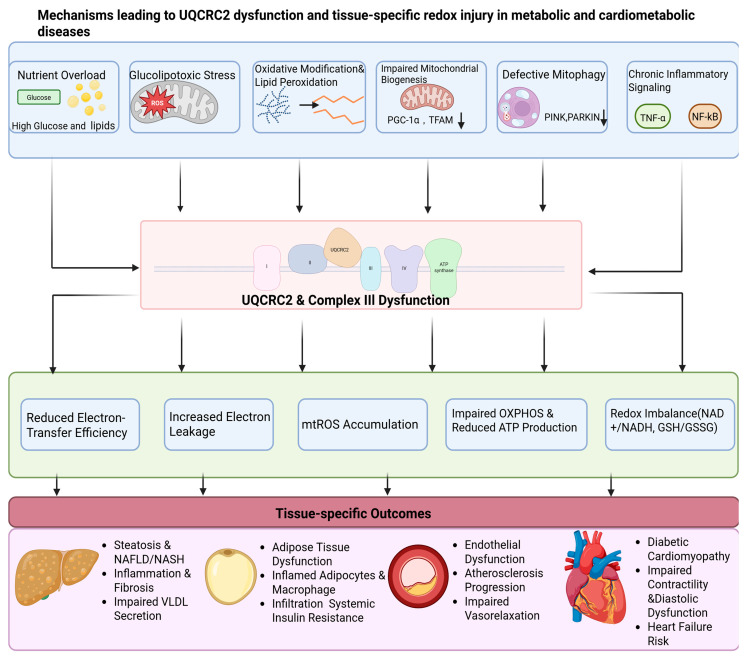
Mechanisms leading to UQCRC2 dysfunction and tissue-specific redox injury in metabolic and cardiometabolic diseases. Nutrient overload, glucolipotoxic stress, oxidative modification and lipid peroxidation, impaired mitochondrial biogenesis, defective mitophagy, and chronic inflammatory signaling may converge on UQCRC2-containing mitochondrial Complex III. These insults can impair Complex III assembly and electron-transfer efficiency, thereby promoting electron leakage, mtROS accumulation, defective OXPHOS, reduced ATP production, and disruption of mitochondrial redox balance, including altered NAD^+^/NADH and glutathione homeostasis. The resulting mitochondrial redox dysfunction contributes to tissue-specific pathological outcomes, including hepatic steatosis, inflammation, fibrosis, and impaired VLDL secretion in the liver; adipose tissue dysfunction, adipocyte inflammation, macrophage infiltration, and systemic IR in adipose tissue; endothelial dysfunction, atherosclerosis progression, and impaired vasorelaxation in the vasculature; and diabetic cardiomyopathy, impaired myocardial contractility and diastolic function, and increased heart failure risk in the heart. Created in BioRender. Chen, S. (2026) https://BioRender.com/ic8gni4, accessed on 10 June 2026.

UQCRC2-dependent mitochondrial redox dysfunction may contribute to metabolic and cardiometabolic diseases. Under nutrient overload, glucolipotoxic stress, oxidative damage, impaired mitochondrial biogenesis, defective mitochondrial quality control, and chronic inflammatory stimulation, UQCRC2 expression or function may become compromised, ultimately linking Complex III dysfunction to IR, hepatic injury, adipose dysfunction, vascular inflammation, and myocardial remodeling (Figure 3).

### 3.1. Mechanisms of UQCRC2 Downregulation and Dysfunction

Before discussing disease-specific contexts, it is important to clarify how UQCRC2 expression or function may become impaired under metabolic stress. Current evidence suggests that UQCRC2 dysregulation may arise from several interconnected mechanisms, including nutrient overload, glucolipotoxic stress, oxidative modification of respiratory-chain proteins, impaired mitochondrial biogenesis, defective mitochondrial quality control, and chronic inflammatory signaling [13].

Nutrient overload and glucolipotoxic stress can increase redox pressure on the mitochondrial electron transport chain. Under conditions of hyperglycemia or lipid excess, increased delivery of NADH and FADH_2_ to the respiratory chain may elevate mitochondrial membrane potential and overload electron-transfer capacity. Because Complex III is an important site of electron transfer and mtROS generation, excessive substrate input can increase the redox burden on Complex III, favoring electron leakage and mtROS overproduction. In this setting, reduced UQCRC2 abundance or impaired assembly of UQCRC2-associated Complex III may further decrease electron-transfer efficiency, thereby amplifying mitochondrial redox imbalance and OXPHOS impairment [9].

Oxidative stress and lipid peroxidation may also impair UQCRC2-related Complex III function. In metabolically stressed tissues, excessive mtROS promotes the formation of reactive lipid peroxidation products, including 4-hydroxynonenal (4-HNE) and malondialdehyde (MDA) [14]. These electrophilic aldehydes can covalently modify respiratory-chain proteins, including Complex III components, and thereby affect their conformation, assembly stability, and electron-transfer capacity. This mechanism is particularly relevant in lipotoxic tissues such as the steatotic liver. As a result, Complex III dysfunction can promote mtROS production, while oxidative modifications further compromise UQCRC2-associated Complex III activity and sustain mitochondrial redox injury.

UQCRC2 downregulation may reflect impaired mitochondrial biogenesis and respiratory-chain protein homeostasis. Signaling pathways such as AMPK–SIRT1–PGC-1α support mitochondrial biogenesis, oxidative metabolism, and the expression of OXPHOS-related proteins [15]. Suppression of these pathways under chronic metabolic stress may decline the abundance of ETC proteins and weaken OXPHOS capacity. In parallel, persistent activation of stress-related pathways, including NF-κB and mTORC1 signaling, may disturb mitochondrial proteostasis and mitophagy, impairing the adaptive remodeling of respiratory-chain complexes [16]. These changes may not specifically target UQCRC2 alone, but they can reduce the stability and functional integrity of UQCRC2-containing Complex III.

Defective mitochondrial quality control may aggravate UQCRC2 dysfunction [17]. Under physiological conditions, mitophagy removes mitochondria with impaired respiratory-chain activity before they become persistent sources of mtROS [18]. However, under chronic cardiometabolic stress, impaired mitophagy, disturbed mitochondrial proteostasis, and weakened antioxidant defense can allow damaged mitochondria to accumulate. This can sustain mtROS generation, promote further oxidative damage to Complex III proteins, and reinforce UQCRC2-related mitochondrial dysfunction.

Finally, genetic defects in Complex III subunits or assembly factors show that an intact Complex III is essential for mitochondrial energy production. Pathogenic variants in Complex III subunits or assembly factors, including UQCRC2 and UQCC2, have been reported to cause mitochondrial complex III deficiency, often presenting as early-onset mitochondrial or metabolic disease [19]. However, these rare monogenic disorders should be distinguished from common metabolic and cardiometabolic diseases, in which UQCRC2 dysfunction is more likely to result from acquired glucolipotoxic, inflammatory, and oxidative stress. Whether UQCRC2 variants contribute to susceptibility or disease severity in common cardiometabolic phenotypes remains insufficiently defined.

Overall, UQCRC2 dysfunction may arise from multiple acquired stressors, including glucolipotoxicity, oxidative damage, impaired mitochondrial biogenesis, defective mitophagy, and chronic inflammation [13]. These mechanisms may converge on UQCRC2-containing Complex III, leading to reduced electron-transfer efficiency, mtROS accumulation, OXPHOS impairment, and mitochondrial redox imbalance.

### 3.2. UQCRC2 in T2DM and IR

T2DM is characterized by systemic IR, impaired glucose homeostasis and progressive β-cell dysfunction, conditions tightly coupled to mitochondrial dysfunction [20]. In this disease context, UQCRC2-related Complex III dysfunction may provide a mitochondrial redox mechanism linking glucolipotoxic stress to impaired insulin signaling. When glucotoxic or lipotoxic stress destabilizes UQCRC2-dependent Complex III performance, electron leak increases, mtROS rises, and bioenergetic efficiency declines [9,21]. These changes impair insulin signaling in liver, skeletal muscle, and adipose tissue, linking mitochondrial stress to systemic IR.

Recent studies suggest that downregulation or structural injury of UQCRC2 contributes to the dysfunction of Complex III in T2DM, extending mitochondrial stress to IR phenotypes. For example, transcriptomic analyses of human liver have shown that, in patients with T2DM, downregulation of UQCRC2 and other Complex III components aligns with diminished oxidative metabolic capacity [22]. In cellular and mouse models of IR, oat β-D-glucan treatment restored UQCRC2 expression, leading to reduced mitochondrial oxidative stress and subsequently improved OXPHOS efficiency and hepatic glucose metabolism [23]. Yang et al. reported that the SGLT2 inhibitor (canagliflozin) increased the expression of UQCRC2 in adipocytes, accompanied by improved mitochondrial function and insulin sensitivity [24].

Together, these findings support a mechanistic cascade in which reduced UQCRC2 expression or impaired Complex III function promotes electron leakage, mtROS accumulation, and mitochondrial bioenergetic failure, thereby contributing to impaired insulin signaling. Conversely, interventions that restore UQCRC2-related mitochondrial function may help alleviate redox imbalance and improve metabolic homeostasis.

In sum, UQCRC2 acts as a key regulator of Complex III performance in T2DM, linking oxidative stress to IR. This perspective suggests potential therapeutic avenues that target UQCRC2/Complex III to mitigate mitochondrial dysfunction and improve metabolic outcomes.

### 3.3. UQCRC2 in MASLD and Hepatic Lipotoxic Redox Stress

MASLD is the most prevalent chronic liver disease worldwide and is tightly linked to obesity, dyslipidemia, hypertension, and T2DM. MASLD provides a representative disease context in which lipid overload, oxidative damage, impaired antioxidant defense, and mitochondrial dysfunction converge on Complex III-dependent redox instability. Pathologically, MASLD spans simple steatosis (MASL) to steatohepatitis with hepatocyte ballooning and lobular inflammation (MASH), with a subset progressing to fibrosis and cirrhosis [25]. Given the hepatic dependence on mitochondrial β-oxidation and OXPHOS, defects in the respiratory chain, particularly at Complex III, are well positioned to transduce lipotoxic stress into bioenergetic dysfunction and inflammatory activation [4,26]. In MASLD, lipid overload, lipid peroxidation, impaired antioxidant defense, and mitochondrial injury converge on Complex III-dependent redox instability. Overall, the UQCRC2-dependent Complex III dysfunction mechanistically links hepatic steatosis to bioenergetic impairment, mtROS accumulation, impaired OXPHOS, and inflammatory liver injury.

#### 3.3.1. Role of UQCRC2 in Lipotoxic Redox Stress and Complex III Dysfunction

In steatotic hepatocytes, markers of lipid peroxidation, especially 4-hydroxynonenal and malondialdehyde, accumulate and form covalent adducts with respiratory-chain proteins, including the Complex III core subunit UQCRC2 [27,28]. Such adduction is associated with depressed Complex III activity, increased electron leak, and amplification of mtROS. In parallel, mitochondrial antioxidant defense is weakened in MASLD. Mitochondrial antioxidant capacity declines as mitochondrial glutathione (mGSH) is depleted [29]. It can be exacerbated by cholesterol accumulation in the mitochondrial inner membrane, which limits the transport of cytosolic glutathione (cGSH) into the mitochondrial matrix [30]. These processes suggest that UQCRC2-related Complex III dysfunction may serve as a mitochondrial redox checkpoint through which lipid overload is translated into oxidative injury, impaired antioxidant defense, and progression from simple steatosis to inflammatory liver damage.

#### 3.3.2. Role of UQCRC2 in mtROS-Dependent Inflammasome Activation

In steatotic livers, disruption of UQCRC2-supported Complex III function compromises mitochondrial redox homeostasis, promoting electron leakage and sustained mtROS accumulation [26]. In addition to driving bioenergetic impairment, mtROS acts as a critical inflammatory signal. On the one hand, mtROS promotes transcriptional priming through TLR-NF-κB/AP-1 signaling, enhancing the expression of NLRP3 and pro-IL-1β/IL-18. On the other hand, mitochondrial injury releases danger-associated molecular patterns (DAMPs), including oxidized mtDNA and externalized cardiolipin, which drive NLRP3–ASC complex assembly and subsequent caspase-1 activation [31,32]. Inflammasome activation culminates in gasdermin D (GSDMD) cleavage and pyroptotic cell death, while defective mitophagy further amplifies this process by permitting the accumulation of dysfunctional, ROS-producing mitochondria [33].

Animal, cellular, and human studies were performed to explore the exact mechanism between Complex III dysfunction and inflammasome activation. In the livers of offspring mice exposed to aflatoxin B1, mitochondrial damage and inflammatory activation were accompanied by reduced expression of UQCRC2, reflecting compromised Complex III under toxic stress [34]. Furthermore, transcriptomic network analysis of livers in T2DM patients identified UQCRC2 as a hub gene, pointing to a potential link between diabetic liver pathology and mitochondrial fatty-acid metabolic programs [22]. Therefore, in MASLD, UQCRC2 dysfunction may not only impair mitochondrial energy metabolism but also amplify mtROS-dependent inflammatory signaling, thereby connecting redox imbalance with inflammasome activation and hepatocellular injury.

### 3.4. UQCRC2 in Obesity and Adipose Redox Dysfunction

Obesity is a major global public-health challenge. By 2030, more than one billion adults are projected to be living with obesity and its complications, imposing substantial health and economic burdens. Extensive epidemiological evidence identifies obesity as a principal driver of T2DM and MASLD [35,36]. Obesity is characterized not only by excessive lipid storage but also by progressive adipose tissue dysfunction. Expanded WAT, particularly visceral adipose tissue, functions as an endocrine and immune-active organ that regulates adipokine secretion (e.g., leptin and adiponectin), inflammatory cytokine production (e.g., TNF-α and IL-6), and systemic insulin sensitivity [37,38].

Mitochondrial dysfunction in WAT has emerged as a convergent mechanism linking obesity to systemic metabolic dysregulation. In obesity, WAT commonly exhibits reduced mitochondrial abundance and diminished oxidative metabolism, contributing to impaired fatty-acid oxidation (FAO) and lower energy expenditure [38,39]. In obesity, adipose tissue dysfunction is closely associated with mitochondrial redox imbalance. Although WAT shows relatively lower basal UQCRC2 expression than highly oxidative tissues, adipocytes are sensitive to mitochondrial redox changes. Impaired UQCRC2-dependent Complex III activity may shift adipocyte mitochondrial signaling from physiological ROS-dependent regulation toward sustained oxidative stress, thereby contributing to defective adipocyte differentiation, maladaptive adipose expansion, inflammation, and systemic IR. By maintaining Complex III stability and electron-transfer efficiency, UQCRC2 may help preserve the balance between adaptive mitochondrial signaling and pathological oxidative stress in adipose tissue.

Adipocyte differentiation is an energy-demanding process during which activation of the PPARγ/C/EBPα transcriptional cascade is accompanied by increased mitochondrial biogenesis, oxygen consumption, and fatty-acid oxidation. Consequently, respiratory chain integrity is crucial for maintaining OXPHOS capacity to meet the energetic demands of adipogenic differentiation and maturation. When metabolic stress or pharmacological inhibition disrupts Complex III integrity, it declines electron-transfer efficiency, resulting in sustained mtROS elevation. Excessive mtROS disturbs redox-dependent regulators within the PPARγ/C/EBPα network and impairs lipid-droplet formation, ultimately suppressing adipocyte differentiation [40]. Experimental evidence supports the involvement of Complex III-derived ROS in adipocyte differentiation. Pharmacological inhibition of Complex III with agents such as antimycin A increases mtROS and markedly blocks adipogenic progression [41]. Conversely, maintaining the integrity of Complex III and activating the AMPK–PGC-1α axis preserves mitochondrial biogenesis and oxidative capacity, while mitophagy limits mtROS and maintains OXPHOS during adipogenic differentiation [42]. In contrast, disrupted UQCRC2-dependent Complex III function reduces electron transfer efficiency, leading to impaired FAO and limited ATP availability. This deficit, together with elevated mtROS, undermines normal adipocyte differentiation. Metabolic reprogramming under these conditions biases adipose expansion toward hypertrophic enlargement rather than hyperplastic recruitment of new adipocytes, producing an unfavorable adipokine milieu and heightened inflammation, which ultimately impairs adipogenesis and promotes systemic IR and cardiometabolic risk [38].

Overall, these findings position UQCRC2 as a key bioenergetic regulator ynt linking between adipose mitochondrial redox balance, adipocyte differentiation, and systemic insulin resistance in obesity.

### 3.5. UQCRC2 in CVDs

Mitochondrial dysfunction linked to UQCRC2 in the liver, pancreas, and adipose tissue disrupts metabolic homeostasis, giving rise to systemic abnormalities, including IR, hyperglycemia, dyslipidemia, and low-grade inflammation. The cardiovascular system is particularly susceptible because cardiomyocytes and vascular endothelial cells depend on continuous mitochondrial ATP production to sustain excitation–contraction coupling and vascular homeostasis [43]. Within this metabolic milieu, defects in the respiratory chain—especially impaired assembly and electron transfer at Complex III and its scaffold subunit UQCRC2—provide a clear mechanistic connection between metabolic stress and vascular or myocardial injury. Mitochondrial dysfunction promotes the initiation and progression of CVD through convergent pathways—energetic derangement, redox-mediated endothelial dysfunction, activation of innate immune/inflammatory signaling, and maladaptive structural remodeling—thereby linking mitochondrial impairment to clinical cardiovascular phenotypes [44,45].

#### 3.5.1. Role of UQCRC2 in Vascular Inflammatory Dysfunction

In healthy vessels, endothelial nitric oxide synthase (eNOS) generates nitric oxide (NO), which activates soluble guanylyl cyclase in vascular smooth muscle cells, promotes vasodilation and maintains an antithrombotic, anti-inflammatory phenotype. Under cardiometabolic stress, excess mtROS originating from an overloaded electron-transport chain and structurally compromised Complex III/UQCRC2 reacts with NO to form peroxynitrite, oxidizes tetrahydrobiopterin and promotes eNOS uncoupling [46,47]. NO bioavailability falls, superoxide production increases, and endothelium-dependent vasodilation is impaired. Concurrently, Complex III-driven mtROS activates redox-sensitive transcription factors (NF-κB and AP-1), upregulating adhesion molecules (VCAM-1, ICAM-1) and chemokines that recruit leukocytes to the vascular wall [48]. Upon infiltrating the intima, monocytes engulf modified lipids to differentiate into foam cells. These cells secrete pro-inflammatory cytokines (TNF-α, IL-6, MCP-1), which not only reinforce endothelial dysfunction but also serve as the priming signal for the NLRP3 inflammasome. Subsequently, mitochondrial DAMPs and lipid-derived danger signals—such as oxidized mtDNA, externalized cardiolipin, and oxidized LDL—trigger the assembly of the NLRP3 complex, caspase-1 activation, and the final release of IL-1β/IL-18 [13,31,49,50]. These processes accelerate atherogenesis, enlarge the necrotic core, and weaken the fibrous cap, increasing plaque vulnerability and thrombosis risk. In the vasculature, impaired Complex III/UQCRC2 function contributes to a shift from NO-dominant vasoprotective signaling to mtROS-driven endothelial dysfunction and chronic vascular inflammation. Thus, vascular UQCRC2/Complex III dysfunction may promote a shift from NO-mediated vasoprotection to mtROS-driven endothelial activation, inflammatory recruitment, and atherogenic remodeling.

#### 3.5.2. Role of UQCRC2 in Myocardial Bioenergetics and Ischemia–Reperfusion Injury

In the myocardium, UQCRC2-dependent Complex III activity is essential for sustaining OXPHOS and the tight coupling between mitochondrial ATP production and excitation–contraction. Oxidative modification or assembly defects that impair UQCRC2 function attenuate electron transfer through Complex III, resulting in a reduced proton motive force and ATP synthesis, perturbation of the mitochondrial NADH/NAD^+^ balance, and enhanced mtROS production [51,52,53]. These abnormalities reduce myocardial energetic reserve and make contractile function more vulnerable to metabolic or ischemic stress.

Excess mtROS and ATP deficits jointly disrupt Ca^2+^ handling. Oxidative modifications of SERCA2a and phospholamban impair Ca^2+^ reuptake into the sarcoplasmic reticulum, whereas oxidation of ryanodine receptors promotes abnormal Ca^2+^ release and diastolic Ca^2+^ leak. In parallel, oxidation-induced activation of CaMKII and enhanced mitochondrial Ca^2+^ uptake further promote Ca^2+^ overload [54,55]. This ionic imbalance, coupled with energetic exhaustion, triggers afterdepolarizations, increasing susceptibility to arrhythmia and contractile dysfunction.

Under physiological conditions, such damage would be mitigated by AMPK-driven mitophagy. However, under chronic cardiometabolic stress, persistent mTORC1 activation and depletion of mGSH impair mitochondrial clearance [53,56]. Ultimately, the convergence of energy deficit, oxidative stress, and Ca^2+^ overload provokes the opening of the mitochondrial permeability transition pore (mPTP). Sustained mPTP opening triggers cytochrome-c release and caspase activation, precipitating necrotic or apoptotic cardiomyocyte loss [51,57].

Crucially, these mechanisms are markedly amplified during ischemia–reperfusion. Reperfusion abruptly restores oxygen supply, which can intensify electron leakage, mtROS bursts, Ca^2+^ overload, and mPTP opening. Oxidative modification or instability of UQCRC2-associated Complex III may further impair electron transfer and aggravate post-ischemic mitochondrial dysfunction. Thus, UQCRC2-related Complex III dysfunction may connect bioenergetic failure, redox imbalance, and Ca^2+^ dysregulation with cardiomyocyte death during ischemia–reperfusion injury.

#### 3.5.3. Role of UQCRC2 in Fibrosis and Structural Remodeling

Over time, the combination of endothelial dysfunction, vascular inflammation and cardiomyocyte loss culminates in fibrosis and adverse remodeling, central determinants of heart failure. ROS and inflammatory cytokines generated downstream of Complex III/UQCRC2 dysfunction activate TGF-β/Smad signaling, enhancing synthesis and deposition of extracellular matrix (ECM) proteins such as collagen [58,59,60]. In the vasculature, ECM accumulation promotes arterial stiffness and luminal narrowing, contributing to hypertension and atherosclerosis progression [61,62]; in the myocardium, interstitial and perivascular fibrosis reduce ventricular compliance and impair both systolic and diastolic function [58,59].

Chronic inflammation further shapes the ECM microenvironment. Immune cells recruited to the vessel wall and myocardium release matrix metalloproteinases that remodel the ECM, destabilize atherosclerotic plaques and foster scar formation after myocyte death [63]. Meanwhile, impaired mitophagy—secondary to UQCRC2-related mitochondrial dysfunction—fails to restrain ROS accumulation, sustaining profibrotic signaling [64].

Collectively, these data support a unified model in which UQCRC2-dependent Complex III integrity acts as an upstream regulator of cardiovascular health. Loss of UQCRC2 function enhances mtROS production, disrupts NO and Ca^2+^ signaling, promotes inflammation, lowers the threshold for cell death and drives fibrosis and structural remodeling, thereby linking metabolic stress to clinical cardiovascular phenotypes.

## 4. Translational Evidence, Redox-Based Interventions, and Future Directions

Although most evidence for UQCRC2 function derives from experimental models, a small but growing body of clinical and translational data points to its relevance in human metabolic and cardiovascular disease. Across obesity, T2DM, and MASLD, a common upstream driver is mitochondrial dysfunction in energy and redox control, which links metabolic stress to vascular dysfunction and cardiac vulnerability. Within this framework, Complex III and its scaffold subunit UQCRC2 emerge as a recurrent molecular node rather than a mere downstream by-product of metabolic stress.

Several pharmacological and nutritional interventions have been associated with changes in UQCRC2 expression or UQCRC2-related mitochondrial pathways. Studies of human adipose tissue have demonstrated that the inflammatory microenvironment exerts substantial regulatory effects on mitochondrial function. Pro-inflammatory macrophages enhance adipocyte energy expenditure, whereas anti-inflammatory macrophages downregulate UQCRC2 expression and reduce ATP generation, underscoring the close relationship between immune regulation and complex III activity [65]. In addition, pharmacological interventions provide indirect evidence for the modulation of UQCRC2. The sodium-glucose cotransporter 2 (SGLT2) inhibitor, widely used in clinical practice for T2DM, has been shown to promote mitochondrial remodeling in adipocytes via the AMPK–Sirt1–PGC-1α signaling pathway, leading to upregulation of complex proteins, including UQCRC2, thereby improving energy homeostasis [24]. Randomized controlled trials in peripheral artery disease (PAD) further support the clinical relevance of UQCRC2. Long-term supplementation with cocoa flavanols significantly improved walking performance in patients with PAD, accompanied by increased skeletal muscle expression of UQCRC2 and activation of the Nrf2 antioxidant pathway [66]. These findings are consistent with the view that enhancing complex III function is associated with improved clinical outcomes in patients with PAD. In the cardiovascular field, ischemia–reperfusion injury has been shown to induce oxidative modifications of multiple complex III subunits, including UQCRC2, leading to impaired electron transport and mitochondrial dysfunction. Such mechanisms may account, at least in part, for residual myocardial injury following reperfusion therapy, highlighting UQCRC2 as a potential target for cardioprotection [67].

**Figure 4:**
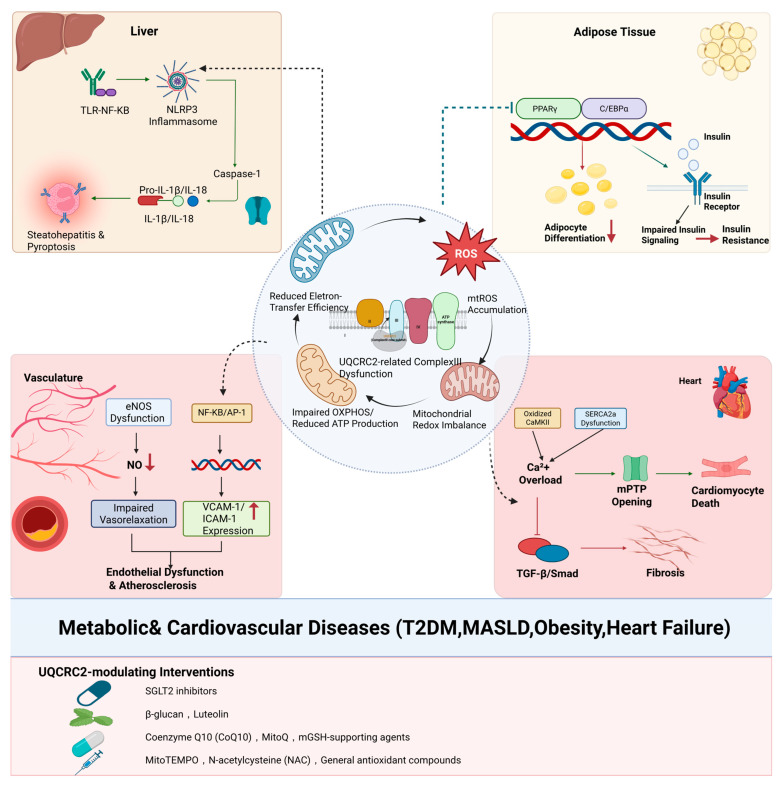
UQCRC2-related mitochondrial dysfunction in metabolic and cardiovascular diseases. UQCRC2-related Complex III dysfunction reduces electron-transfer efficiency, impairs OXPHOS and ATP production, and promotes mtROS accumulation and mitochondrial redox imbalance. These changes may contribute to tissue-specific injury, including inflammasome activation and pyroptosis in the liver, impaired adipocyte differentiation and insulin resistance in adipose tissue, endothelial dysfunction and atherosclerosis in the vasculature, and Ca^2+^ overload, mPTP opening, cardiomyocyte death, and fibrosis in the heart. Potential UQCRC2-modulating interventions include SGLT2 inhibitors, β-glucan, luteolin, coenzyme Q10, mitochondria-targeted antioxidants, N-acetylcysteine, and other antioxidant compounds. Solid arrows indicate major mechanistic flows, dashed arrows indicate indirect or putative associations, blunt-ended lines indicate inhibition, circular arrows indicate self-amplifying cycles, and red downward arrows indicate decreased activity or function. Created in BioRender. Chen, S. (2026) https://BioRender.com/7c2260a, accessed on 10 June 2026.

In addition to pharmacological agents, several natural compounds have been reported to modulate UQCRC2-related pathways. For example, luteolin has been shown to partially restore hepatic UQCRC2 expression in an obese PCOS rat model and concurrently improve metabolic dysfunction. These findings suggest that luteolin may confer metabolic benefits through regulation of the UQCRC2/PI3K–AKT axis [68]. Yuan et al. reported that UQCRC2-associated Complex III integrity is pharmacologically modifiable under cardiometabolic stress. In hypoxia, curcumin treatment increased the abundance of UQCRC2 along with other ETC components and was linked to improvements in mitochondrial stress, supporting the concept that stabilizing Complex III protein homeostasis may contribute to mitochondrial protection in ischemic contexts [69]. Despite these advances, current evidence remains preliminary and is largely derived from cellular and animal models, with limited clinical validation. The clinical application of UQCRC2 as a biomarker or therapeutic target also remains challenging. UQCRC2 abundance could theoretically be assessed in peripheral blood mononuclear cells, skeletal muscle biopsies, adipose tissue biopsies, liver samples, or cardiovascular tissues obtained in specific clinical settings [70]. Functional readouts, such as Complex III activity, OXPHOS capacity, mtROS production, and redox-related markers, may provide additional information on the consequences of UQCRC2 alteration [71]. However, standardized and clinically validated assays for measuring UQCRC2 expression, UQCRC2-containing Complex III integrity, or Complex III-centered redox dysfunction remain limited in routine practice. Tissue biopsies provide disease-relevant information but are invasive, whereas peripheral blood-based assays are more accessible but may not fully reflect tissue-specific mitochondrial dysfunction in the liver, adipose tissue, skeletal muscle, or myocardium. Taken together, UQCRC2 emerges as a potential molecular link between mitochondrial dysfunction and systemic metabolic derangement. Future studies integrating patient cohorts, clinical assays, tissue-specific models, and multi-omics analyses will be essential to define its clinical relevance, biomarker value, and therapeutic feasibility. At present, UQCRC2-centered strategies should focus on preserving Complex III integrity, restoring mitochondrial bioenergetic and redox homeostasis, and developing redox-based interventions for metabolic and cardiometabolic diseases (Figure 4).

## 5. Conclusions

Metabolic diseases such as T2DM, MASLD, obesity, and cardiovascular disorders are closely linked to mitochondrial dysfunction. UQCRC2, a core subunit of complex III, plays a critical role in electron transport and mitochondrial redox homeostasis, therefore supporting OXPHOS. UQCRC2 deficiency impairs OXPHOS and increases ROS levels. These changes exacerbate IR and promote lipotoxic injury, adipose dysfunction, and cardio-hepatic inflammation with fibrotic remodeling. These alterations highlight UQCRC2 as an important mediator connecting mitochondrial dysfunction with systemic metabolic and cardiovascular complications.

In summary, UQCRC2 emerges as a redox-sensitive mitochondrial node linking Complex III dysfunction to mtROS accumulation, impaired antioxidant defense, glucose–lipid dysregulation, inflammatory activation, and cardiometabolic injury. Although current evidence supports the biological relevance of UQCRC2 in metabolic and cardiovascular disorders, its causal role and clinical utility are yet to be fully established. Future studies should integrate patient-based evidence, tissue-specific models, multi-omics analysis, and targeted redox interventions to determine whether UQCRC2-centered mitochondrial modulation can be translated into clinically useful biomarkers or therapeutic strategies.

## Data Availability

No new data were created or analyzed in this study.

## Abbreviations

The following abbreviations are used in this manuscript:

CVD	Cardiovascular disease
ETC	Electron transport chain
FAO	Fatty acid oxidation
IR	Insulin resistance
MASLD	Metabolic dysfunction-associated steatotic liver disease
MASH	Metabolic dysfunction-associated steatohepatitis
MS	Metabolic syndrome
mtROS	Mitochondrial reactive oxygen species
mPTP	Mitochondrial permeability transition pore
NLRP3	NOD-like receptor family pyrin domain containing 3
OXPHOS	Oxidative phosphorylation
T2DM	Type 2 diabetes mellitus
TCA	Tricarboxylic acid
UQCRC2	Ubiquinol–cytochrome c reductase core protein 2
WAT	White adipose tissue
Δψm	Mitochondrial membrane potential
